# No more business as usual: Agile and effective responses to emerging pathogen threats require open data and open analytics

**DOI:** 10.1371/journal.ppat.1008643

**Published:** 2020-08-13

**Authors:** Dannon Baker, Marius van den Beek, Daniel Blankenberg, Dave Bouvier, John Chilton, Nate Coraor, Frederik Coppens, Ignacio Eguinoa, Simon Gladman, Björn Grüning, Nicholas Keener, Delphine Larivière, Andrew Lonie, Sergei Kosakovsky Pond, Wolfgang Maier, Anton Nekrutenko, James Taylor, Steven Weaver

**Affiliations:** 1 Johns Hopkins University, Baltimore, Maryland, United States of America; 2 The Pennsylvania State University, University Park, Pennsylvania, United States of America; 3 Cleveland Clinic, Cleveland, Ohio, United States of America; 4 VIB Center for Plant Systems Biology, Ghent, Belgium; 5 Department of Plant Biotechnology and Bioinformatics, Ghent University, Ghent, Belgium; 6 University of Melbourne, Melbourne, Australia; 7 Queensland Cyber Infrastructure Foundation, St. Lucia, Australia; 8 University of Freiburg, Freiburg im Breisgau, Germany; 9 Temple University, Philadelphia, Pennsylvania, United States of America; University of Pittsburgh, UNITED STATES

## Abstract

The current state of much of the Wuhan pneumonia virus (severe acute respiratory syndrome coronavirus 2 [SARS-CoV-2]) research shows a regrettable lack of data sharing and considerable analytical obfuscation. This impedes global research cooperation, which is essential for tackling public health emergencies and requires unimpeded access to data, analysis tools, and computational infrastructure. Here, we show that community efforts in developing open analytical software tools over the past 10 years, combined with national investments into scientific computational infrastructure, can overcome these deficiencies and provide an accessible platform for tackling global health emergencies in an open and transparent manner. Specifically, we use all SARS-CoV-2 genomic data available in the public domain so far to (1) underscore the importance of access to raw data and (2) demonstrate that existing community efforts in curation and deployment of biomedical software can reliably support rapid, reproducible research during global health crises. All our analyses are fully documented at https://github.com/galaxyproject/SARS-CoV-2.

The initial publications describing genomic features of SARS-CoV-2 [1–4] used Illumina and Oxford nanopore data to elucidate the sequence composition of patient specimens (although only Wu and colleagues [3] explicitly provided the accession numbers for their raw short-read sequencing data). However, their approaches to processing, assembly, and analysis of raw data differed widely (Table 1) and ranged from transparent [3] to entirely opaque [4]. Such lack of analytical transparency sets a dangerous precedent. Infectious disease outbreaks often occur in locations where infrastructure necessary for data analysis may be inaccessible or unbiased interpretation of results may be politically untenable. As a consequence, there is a global need to ensure access to free, open, and robust analytical approaches that can be used by anyone in the world to analyze, interpret, and share data. Can existing tools and computational resources support such a global need? Here, we show that they can: we analyzed all available raw SARS-CoV-2 data to demonstrate that analyses described in [1–4] can be reproduced on public infrastructure using open-source tools by any researcher with an internet connection.

**Table 1:** Methods used for the analysis of primary SARS-CoV-2 data.

Analysis stage	Publication			
	[3]	[1]	[2]	[4]
Tools	Bowtie2 MegaHit Trinity MAFFT PhyML MEGA RDP4	BWA Geneous MegaHit MAFFT Clustal RAxML	BWA SPAdes CNCBio MAFFT RAxML	minimap Sequencher FreeBayes?
Versions specified	+	+	+	+
Parameters specified	−	−	−	−
Raw data	+	?	−	−

? = uncertain (e.g., Holshue and colleagues [4] identify FreeBayes as an assembly tool).

Abbreviation: SARS-CoV-2, severe acute respiratory syndrome coronavirus 2

We exclusively used free software tools publicly available from the BioConda package distribution system [5], which were deployed through the worldwide network of open Galaxy platforms [6] and executed using public high-throughput computational infrastructure (XSEDE in the United States, VSC in Belgium, de.NBI, and ELIXIR in the European Union, NeCTAR Research Cloud in Australia). We also used an open-source Jupyter environment [7] for exploratory analysis of data. All analyses performed here are fully documented and accessible at https://github.com/galaxyproject/SARS-CoV-2/ and https://doi.org/10.5281/zenodo.3685264 (note that these are being continuously updated).

We divided our analysis into the following stages: (1) read preprocessing, (2) genome assembly, (3) timing the most recent common ancestor (MRCA), (4) analysis of genomic variation within individual samples, and (5) recombination and selection analyses.

We preprocessed currently available (as of February 19, 2020) sequencing read data sets for SARS-CoV-2 (S1 Table) by removing adapter contamination and reads derived from human transcripts and combined the resulting data sets. This was done to SARS-CoV-2–specific reads, which constitute only a fraction of the original data. These were used as inputs for SPAdes assembler [8] and Unicycler [9]—an assembly pipeline based on SPAdes that includes a number of preprocessing and polishing steps. Both approaches were able to reconstruct a full-length SARS-CoV-2 genome, with Unicycler producing a cleaner assembly graph. Its largest contig (29,781 bp) had 100% identity to the published assembly NC_045512.

Next, we estimated the date of the MRCA of SARS-CoV-2. For this, we used simple root-to-tip regression [10] (more complex and powerful phylodynamics methods could certainly be used, but for these data with very low levels of sequence divergence, simpler and faster methods suffice). Using a set of sequences (we removed identical genomes by retaining only one representative from a set of identical sequences) from all SARS-CoV-2 sequences available as of February 16, 2020, we obtained an MRCA date of October 24, 2019, which is close to other existing estimates [11].

The vast majority of SARS-CoV-2 genomic data available at the time of writing are partially or fully assembled genomes. There is no public access to sequence reads that were used to produce these assemblies: as of February 19, 2020—more than 2 months since the beginning of the outbreak—there were only six raw data sets (S1 Table).

This should be unacceptable because raw read data can be used to uncover viral diversity within individual samples and evaluate robustness and reliability of the assembly. To demonstrate that such diversity exists, we mapped Illumina reads against SARS-CoV-2 reference (NC_045512) and identified sequence variants with frequencies above 5% while taking into account quality of alternative bases and strand bias. Five percent was selected as a conservative threshold that can be reliably resolved from Illumina data [12]. Using this threshold, 39 single nucleotide variants (SNVs) were identified in total across all samples (Fig 1). The most prominent sequence variant was observed in sample SRR10903401. It is an A-to-C substitution with alternate allele frequency of 38% that causes a Lys^921^Gln amino acid replacement within the spike glycoprotein S (product of gene *S*). S is a homotrimeric protein containing S1 and S2 subdomains, which mediate receptor recognition and membrane fusion, respectively [13]. S2 subdomains contain two heptad repeat (repeats of units containing seven amino acids) regions: HR1 and HR2. The Lys^921^Gln substitution we observed is located in HR1 and forms a salt bridge with Gln^1188^ within HR2. This is one in a series of salt bridges involved in the formation of the HR1/HR2 hairpin structures [14]. This site invariably contains Lys in all human severe acute respiratory syndrome (SARS)-related coronaviruses (S protein residue 903) as well as in many other coronaviruses (Fig 2). However, more distantly related coronaviruses, including transmissible gastroenteritis coronavirus (TGEV), the porcine respiratory coronavirus (PRCV), the canine coronavirus (CCV), the feline peritonitis virus (FIPV), and the porcine epidemic diarrhea virus (PDEV), all contain Gln at the corresponding position ([14] and Fig 2). The Lys^921^Gln change would prevent the formation of the salt bridge with Gln^1188^ and may have structural and functional implications for the spike protein structure and, consequently, SARS-CoV-2 virulence. This potentially adaptive change was not observed in the other two samples, and a lack of raw read data prevented us from identifying it in other geographically and temporally distributed samples.

**Fig 1:**
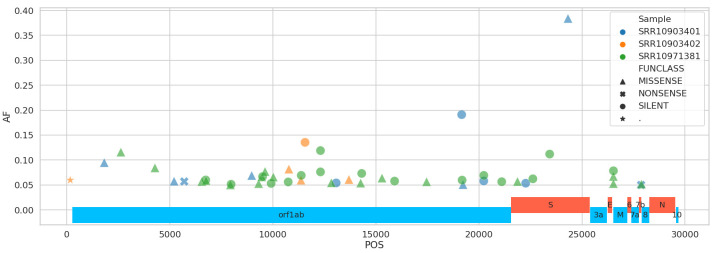
Distribution of nucleotide changes across SARS-CoV-2 genome. AF, minor allele frequency; POS, position; SARS-CoV-2, severe acute respiratory syndrome coronavirus 2.

**Fig 2:**
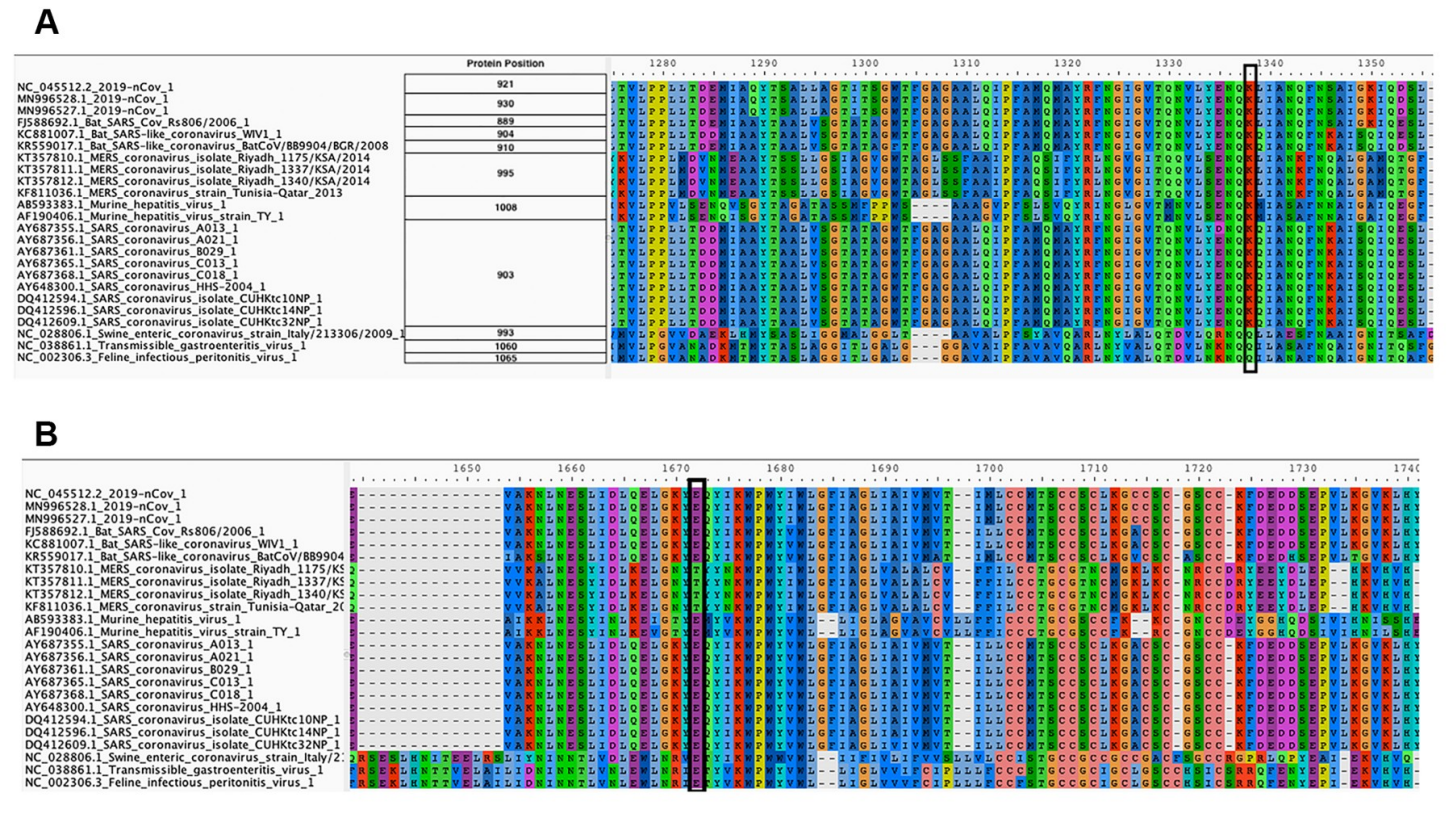
Amino acid alignment of spike glycoprotein regions HR1 (A) and HR2 (B). The site of the Lys^921^Gln substitution observed by us in a SARS-CoV-2 isolate is highlighted with a black rectangle in panel A. Its corresponding salt bridge partner is highlighted with a black rectangle in panel B. SARS-CoV-2, severe acute respiratory syndrome coronavirus 2.

To detect potential genome rearrangement events that might have led to the emergence of SARS-CoV-2, we performed analysis of recombination using a genetic algorithm approach [15]. Wu and colleagues [3] identified two potential recombination breakpoints within the SARS-CoV-2 *S* gene with some segments having higher similarity to Bat ZC45 and ZXC21 coronaviruses (accessions MG772933 and MG772934, respectively), whereas others were more similar to SARS Tor2 and SZ3 isolates (accessions AY274119 and AY304486). Our attempt at reproducing this analysis did identify a set of potential breakpoints similar to the ones reported by Wu and colleagues [3], but they lacked robust statistical support (Fig 3).

**Fig 3:**
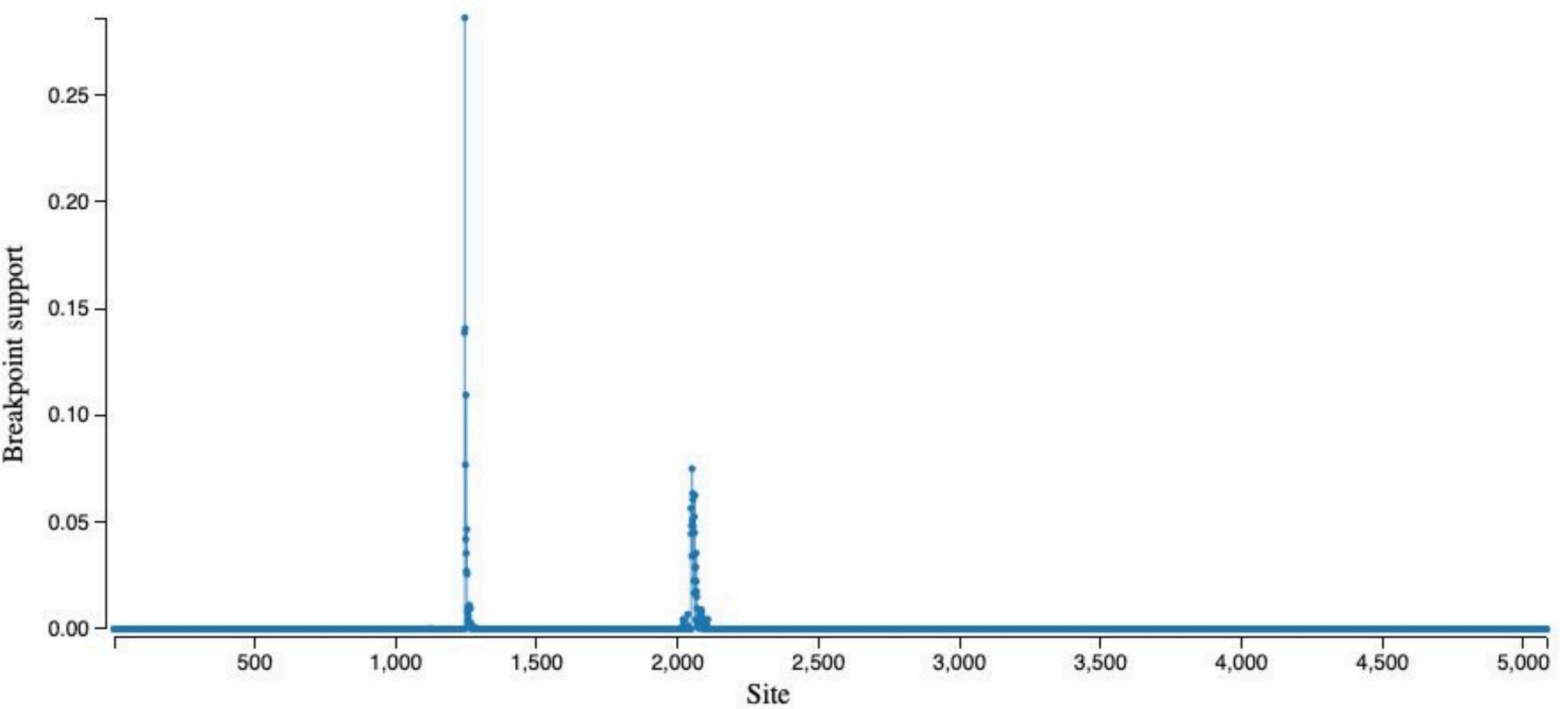
Location of potential recombination breakpoints along the *S* gene (GARD analysis).

Finally, we performed a branch-level test for positive selection on a codon alignment of the *S* gene from SARS-CoV-2, SARS-Tor2, as well as Bat ZC45, ZXC21, and Rp3 coronaviruses, specifically to identify whether there was any evidence of diversifying selection along the ancestral branch leading to SARS-CoV-2 isolates. We found statistically significant evidence of positive diversifying selection (approximately 7% of *S* gene sites) along the branch leading to SARS-CoV-2 (Fig 4).

**Fig 4:**
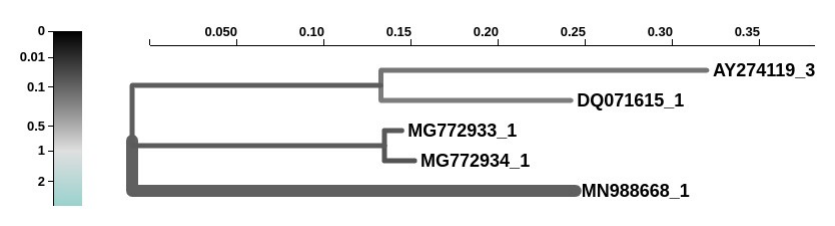
Analysis of branch-specific positive diversifying selection (aBSREL) along the branch leading to SARS-CoV-2 (MN988688). SARS-CoV-2, severe acute respiratory syndrome coronavirus 2.

The goal of our study was to (1) raise awareness of the lack of primary data necessary to effectively respond to global emergencies such as the coronavirus disease 2019 (COVID-19) outbreak and (2) demonstrate that all analyses can be performed transparently with already-existing open-source publicly available tools and computational infrastructure. The first problem—reluctance to share primary data—has its roots in the fact that the ultimate reward for research efforts is a highly cited publication. As a result, individual researchers are naturally averse to sharing primary data prior to manuscript acceptance. The second issue—underutilization of existing, community-supported tools and analysis frameworks—may be due to the lack of sustained efforts to educate the biomedical community about appropriate practices in (genomic) data analysis. Such efforts exist (e.g., [16]) but have difficulties reaching a wide audience because prominent scientific publication outlets are reluctant to accept data analysis tutorials or reviews. Yet the only way to improve accessibility and reproducibility of biomedical research is through dissemination of appropriate analysis practices.

We want to particularly emphasize the issue of irreproducibility. All researchers involved in any given outbreak research should have access to a set of community-curated tools in the same way as they have access to SARS-CoV-2 real-time PCR (RT-PCR) primers [17]. Moreover, they should have access to computational infrastructure that can execute these tools and apply them to potentially large next-generation sequencing (NGS) data sets. This is essential because precious time is spent on “reinventing the wheel.” Instead, in an ideal world, after reading any of the original SARS-CoV-2 manuscripts, any researcher should be able to apply the same analytical procedures to their own data. To illustrate these points, we assessed the reproducibility of the four initial manuscripts describing the SARS-CoV-2 genome (S1 Table). All manuscripts reported versions of the software used, but none listed parameters used. This effectively prevents quality control and replication because outcomes of complex procedures such as genome assembly, phylogenetic reconstruction, and recombination analysis are notoriously parameter dependent. One of the manuscripts [4] explicitly lists FreeBayes [18], a variant discovery tool, as software used for short-read assembly—something that FreeBayes is not capable of doing. Finally, only [3] provided access to the raw data, rendering the other three manuscripts unverifiable and irreproducible.

Our short study demonstrates that viral genome analyses can be performed using open worldwide scientific infrastructure that relies entirely on community-curated and -supported open-source software. Although we used Galaxy as the platform to execute all analyses described here, the individual software components can be obtained directly from BioConda and run independently. They can be combined into workflows using systems like the Common Workflow Language (CWL) [19], Nextflow [20], or Snakemake [21]. Whatever the execution environment or workflow engine, using community-supported, versioned, open-source tools makes data analyses robust and transparent. This increases the quality, efficiency, and ultimately, impact of biomedical research.

In an age of digital connectedness, open, highly accessible, globally shared data and analysis platforms have the potential to transform the way biomedical research is done, opening the way to “global research markets” in which competition arises from deriving understanding rather than access to samples and data. Other disciplines have embraced the benefits of global data generation and sharing—astronomy and high-energy physics being two highly successful examples. We have the opportunity to mirror their successes in infrastructure funding by demonstrating that biological research can embrace the same global perspective on common infrastructure investment and data sharing.

## Changes during review process

The review and publication process of the original version of this manuscript took approximately 5 months. During this time the number of available data sets increased significantly (see https://covid19.galaxyproject.org for the latest results). As of July 31, 2020, the number of available raw read data sets for SARS-CoV-2 was 28,973—a significant increase over the number described in the beginning of this manuscript. However, it is still a fraction of the data: there were ~75,000 assembled genomes in GISAID on the day the proofs were submitted. Until the raw data are released, we will have no ability to validate available assemblies, and any talk about unobstructed analysis of SARS-CoV-2 has no merit. This is not the way serious science is done.

## Methods

Our data and analyses are constantly updated. Exact methods and tool versions are available from https://covid19.galaxyproject.org. This repository contains six directories, corresponding to the analyses we have performed: (1) data preprocessing, (2) genome assembly, (3) estimation of MRCA timing, (4) analysis of intrahost variation, (5) analysis of substitutions within the *S* gene, and (6) analysis of recombination and selection. Every page begins with links to workflows and histories at four Galaxy instances in the US (http://usegalaxy.org), EU (http://usegalaxy.eu), Belgium (http://usegalaxy.be), and Australia (http://usegalaxy.org.au). Exact versions of tools used in the analysis are provided at the end of each section.

## Supporting information

S1 Table Raw SARS-CoV-2 sequencing data available at the time of writing (Feb 20, 2020). *Indicates that data may not be reliable (for example, the link between SRR10903402 and [1] is inferred: neither the SRA record nor the manuscript establishes this relationship). On February 21, 2020 new human data sets SRR11092056, SRR11092057, SRR11092058, and SRR11092064 were added to the SARS-CoV-2 archive [22]. Our analyses indicate that these data sets contain no useful SARS-CoV-2 data [23]. BALF, bronchoalveolar lavage fluid; SARS-CoV-2, severe acute respiratory syndrome coronavirus 2; SRA, Sequence Read Archive.
(RTF)
