# Supplementary material for: No more business as usual: Agile and effective responses to emerging pathogen threats require open data and open analytics
Source: PLoS Pathog. 2020 Aug 13;16(8):e1008643. doi: 10.1371/journal.ppat.1008643 (PMC7425854; doi:10.1371/journal.ppat.1008643)
Supplement: S1 Table — *Indicates that data may not be reliable (for example, the link between SRR10903402 and [1] is inferred: neither the SRA record nor the manuscript establishes this relationship). On February 21, 2020 new human data sets SRR11092056, SRR11092057, SRR11092058, and SRR11092064 were added to the SARS-CoV-2 archive [22]. Our analyses indicate that these data sets contain no useful SARS-CoV-2 data [23]. BALF, bronchoalveolar lavage fluid; SARS-CoV-2, severe acute respiratory syndrome coronavirus 2; SRA, Sequence Read Archive. (RTF) [file ppat.1008643.s001.rtf]

013f
#	Dataset	Reads	Source	Technology	Reference	Locality	
1	SRR10903401	476.6K	BALF RNA	Miseq, PE	* [1]	Wuhan	
2	SRR10903402	676.7K	BALF RNA	Miseq, PE	* [1]	?	
3	SRR10971381	28.3M	BALF RNA	Miseq, PE	[3]	?	
4	SRR10948550	425.7K	* RNA	Minion 	?	?	
5	SRR10948474	505.5K	* RNA	Minion 	?	?	
6	SRR10902284	261.9K	* RNA	Minion 	?	?	
